# Supplementary material for: Prevalence and pattern of rheumatic valvular heart disease in Africa: Systematic review and meta-analysis, 2015–2023, population based studies
Source: PLoS One. 2024 Jul 29;19(7):e0302636. doi: 10.1371/journal.pone.0302636 (PMC11285969; doi:10.1371/journal.pone.0302636)
Supplement: S1 Table — (DOCX) [file pone.0302636.s002.docx]

**Supplementary Table 1. Description of studies included in the meta-analysis of the prevalence and pattern of rheumatic heart disease in Africa.**

| First Author name, Year | Country | Sampling unit | Mean Age | RR% | Sample size Included | Prevalence per 1000 | | | Male-RHD* | female-RHD | Prevalence per 1000 of RHD with CI **among | |
| --- | --- | --- | --- | --- | --- | --- | --- | --- | --- | --- | --- | --- |
|  |  |  |  |  |  | RHD with 95% CI | Definite | Borderline |  |  |  |  |
|  |  |  |  |  |  |  |  |  |  |  | Male | Female |
| Tadesse Gemechu et al(2017)[19] | Ethiopia | Community | 13 | 82 | 987 | 56.74(43.95,72.96) | 37.49 | 19.25 | 28 | 28 | 28.37  (19.70,40.70) | 28.37 (19.70, 40.70) |
| Sulafa Ali et al  (2017)[21] | Sudan | Community | 15 | 100 | \| 3315 \| \| --- \| | 61.54 (53.85,70.24) | 40.12 | 21.42 | 96 | 108 | 28.96 (23.77,35.24) | 32.58(27.06, 39.18) |
| Amy Scheel et al  (2018)[22] | Uganda | Community | 20 | 83.6 | 2453 | 24.46(19.05,31.36) | 12.64 | 11.82 | 24 | 36 | 9.78  (6.58, 14.52) | 14.68 (10.62, 20.25) |
| Mark E Engel et al  (2015)[23] | Ethiopia | School | 10.7 | 99 | 2000 | 30.50(23.82,38.98) | 16.50 | 14.00 | – | – | – | – |
| Mark E Engel et al  (2015)[23] | S. Africa | School | 12.2 | 94.3 | 2720 | 20.22(15.57,26.23) | 4.78 | 15.44 | – | – | – | – |
| Mark E Engel et al  (2015)[23] | S. Africa | School | 11.3 | – | 1279 | 12.51(7.715,20.22) | 2.35 | 10.16 | – | – | – | – |
| Mark E Engel et al  (2015)[23] | S. Africa | School | 13.1 | – | 1441 | 27.10(19.86,36.78) | 6.94 | 20.13 | – | – | – | – |
| Ahmed Ali etal (2023)[24] | Egypt | School | 13.1 | 92.86 | 1560 | 23.10(16.72,31.78) | 23.08 | – | 24 | 12 | 15.39  (10.36,22.79) | 7.69  (4.41,13.40 ) |
| Ekanem N. Ekure(  2019)[25] | Nigeria | School | 11.3 | 100 | 4107 | 2.68 (1.496, 4.79) | 0.49 | 2.19 | 7 | 4 | 1.70  (0.83,3.51) | 0.97(0.38,2.50) |
| Aliou Alassane et al(2015)[26] | Senegal | School | 9.7 | 100 | 2019 | 4.95(2.69, 9.09) | 4.95 | – | 6 | 4 | 2.97  (1.36, 6.47) | 1.98(0.77,5.08) |
| Dr.Hailu Abera et al(2016)[20] | Ethiopia | School | 8.86 | 100 | 1874 | 3.20(1.47 6.97) | 3.20 | – | 2 | 4 | 1.07  (0.29,3.88) | 2.13(0.83,5.48) |
| Dejuma Yadeta et al  (2016)[18] | Ethiopia | School | 13.22 | 98.1 | 3238 | 18.22 (14.15, 23.43) | 13.59 | 4.63 | 18 | 26 | 5.56  (3.52, 8.77) | 8.03(5.49, 11.74) |
| J. mucumisti et al(2017)[27] | Rwanda | School | 11.2 | 83.3 | 2501 | 6.80 (4.25 10.86) | 1.60 | 5.20 | 13 | 4 | 5.20  (3.04,8.87) | 1.60(0.62, 4.11) |
| John Musuku et al(2018)[28] | Zambia | School | 15.4 | 100 | 1102 | 11.80(6.91, 20.10 ) | 2.72 | 9.07 | 5 | 8 | 4.54  (1.94,10.58) | 7.26(3.68,14.26) |
| Amy Sims Sanyahumbi etal(2016)[29] | Malawi | School | – | 100 | 1450 | 33.79(25.66, 44.39) | 6.90 | 26.90 | 20 | 29 | 13.79  (8.95, 21.21) | 20(13.96,28.58) |
| Parvina Titus Kazahura et al(2021)[30] | Tanzania | Community | 10.8 | 100 | 949 | 33.72(23.99, 47.21) | 17.91 | 15.81 | 23 | 9 | 24.24  (16.20,36.11) | 9.48(5.0,17.93) |
| Sulafa Ali et al(2018)[15] | Sudan | Community | 10.5 | 100 | 3000 | 2.33(1.13, 4.81) | 0.33 | 2.00 | – | – | 6.01  (3.16,11.38) | 13.35  (8.66, 20.53) |
| Sulafa Ali et al(2018)[15] | Sudan | Community | 10.8 | \| 98.9 \| \| --- \| | 1498 | 19.36 (13.51, 27.67) | 14.69 | 4.67 | 9 | 20 | – | – |
| Sulafa Ali et al(2018)[16] | Sudan | Community | – | 92 | 2129 | 2.35(1.00, 5.49) | 1.88 | 0.47 | – | – | – | – |
| Sulafa Ali et al(2022)[17] | Sudan | Community | – | 100 | 4572 | 10.28(7.74, 13.64) | – | – | – | – | – | – |
| Esin Nkereuwem et al(2020)[31] | Nigeria | School | – | 100 | 417 | 21.58(11.40, 40.50) | 2.40 | 19.19 | 7 | 2 | 16.79 (8.16,34.24) | 4.80(1.32, 17.32) |
| Ujuanbi A. et al(2019)[32] | Nigeria | School | 10.29 | 100 | 461 | 6.51(,2.22 18.96) | 6.51 | – | – | 3 | – | 6.51(2.22,18.96) |
| Pooled prevalence | – | – | – | – | – | 18.41(14.08 to 22.73) | 8.91(6.50,11.3) | 10.69 (7.74 to 13.65) | – | – | 10.16  (6.84 to 13.47) | 9.72(6.46 to 12.98) |

*Rheumatic Heart Disease ** Confidence interval
