# Supplementary material for: Prevalence and pattern of rheumatic valvular heart disease in Africa: Systematic review and meta-analysis, 2015–2023, population based studies
Source: PLoS One. 2024 Jul 29;19(7):e0302636. doi: 10.1371/journal.pone.0302636 (PMC11285969; doi:10.1371/journal.pone.0302636)
Supplement: S1 Appendix — (DOCX) [file pone.0302636.s003.docx]

**Appendix 1: Search strategies**

1. **Google Scholar:**

Search conducted on 01 February, 2024, where possible terms were searched in MeSH as well as text

Record Retrieved=167

All in title: (prevalence* OR magnitude OR Epidemiology) AND ((((Disease, Rheumatic Heart [Title/Abstract]) OR (Diseases, Rheumatic Heart [Title/Abstract])) OR (Heart Disease, Rheumatic [Title/Abstract])) OR (Heart Diseases, Rheumatic [Title/Abstract])) OR (Rheumatic Heart Diseases [Title/Abstract]) AND [Countries name].

1. **Search Conducted in Hinari**

Search conducted 03 February 2024

Possible terms were searched in MeSH as well as text words

Result Retrieved: 396

Summon™: (prevalence* OR magnitude OR Epidemiology) AND ((((Disease, Rheumatic Heart [Title/Abstract]) OR (Diseases, Rheumatic Heart [Title/Abstract])) OR (Heart Disease, Rheumatic [Title/Abstract])) OR (Heart Diseases, Rheumatic [Title/Abstract])) OR (Rheumatic Heart

1. **Search Conducted in Medline (PubMed)**

Search conducted on 04 February, 2024, where possible terms were searched in MeSH as well as text words

Record Retrieved=9

| #4 | ((("prevalence"[Title/Abstract] OR "incidence"[Title/Abstract] OR "magnitude"[Title/Abstract] OR "Epidemiology"[Title/Abstract]) OR (Proportion[Title/Abstract])) AND (((((Disease, Rheumatic Heart[Title/Abstract]) OR (Diseases, Rheumatic Heart[Title/Abstract])) OR (Heart Disease, Rheumatic[Title/Abstract])) OR (Heart Diseases, Rheumatic[Title/Abstract])) OR (Rheumatic Heart Diseases[Title/Abstract]))) AND ((("Algeria"[Title/Abstract] OR "Angola"[Title/Abstract] OR "Benin"[Title/Abstract] OR "Botswana"[Title/Abstract] OR "Burkina Faso"[Title/Abstract] OR "Burundi"[Title/Abstract] OR "Cabo Verde"[Title/Abstract] OR "Cameroon"[Title/Abstract] OR "central African republic"[Title/Abstract] OR "Chad"[Title/Abstract] OR "Comoros"[Title/Abstract] OR "Congo democratic republic of the"[Title/Abstract] OR "Congo republic of the"[Title/Abstract]) OR "cote d ivoire"[Title/Abstract] OR "Djibouti"[Title/Abstract] OR "Egypt"[Title/Abstract] OR "equatorial guinea"[Title/Abstract] OR "Eritrea"[Title/Abstract] OR "Eswatini"[Title/Abstract] OR "Ethiopia"[Title/Abstract] OR "Gabon"[Title/Abstract] OR "Gambia"[Title/Abstract] OR "Ghana"[Title/Abstract] OR "Guinea"[Title/Abstract] OR "Guinea-Bissau"[Title/Abstract] OR "Kenya"[Title/Abstract] OR "Lesotho"[Title/Abstract] OR "Liberia"[Title/Abstract] OR "Libya"[Title/Abstract] OR "Madagascar"[Title/Abstract] OR "Malawi"[Title/Abstract] OR "Mali"[Title/Abstract] OR "Mauritania"[Title/Abstract] OR "Mauritius"[Title/Abstract] OR "Morocco"[Title/Abstract] OR "Mozambique"[Title/Abstract] OR "Namibia"[Title/Abstract] OR "Niger"[Title/Abstract] OR "Nigeria"[Title/Abstract] OR "Rwanda"[Title/Abstract] OR "sao tome and Principe"[Title/Abstract]) OR "Senegal"[Title/Abstract] OR "Seychelles"[Title/Abstract] OR "sierra leone"[Title/Abstract] OR "Somalia"[Title/Abstract] OR "south africa"[Title/Abstract] OR "south sudan"[Title/Abstract] OR "Sudan"[Title/Abstract] OR "Tanzania"[Title/Abstract] OR "Togo"[Title/Abstract] OR "Tunisia"[Title/Abstract] OR "Uganda"[Title/Abstract] OR "Zambia"[Title/Abstract] OR "Zimbabwe"[Title/Abstract]))) | 9 |
| --- | --- | --- |
| #3 | (("Algeria"[Title/Abstract] OR "Angola"[Title/Abstract] OR "Benin"[Title/Abstract] OR "Botswana"[Title/Abstract] OR "burkina faso"[Title/Abstract] OR "Burundi"[Title/Abstract] OR "cabo verde"[Title/Abstract] OR "Cameroon"[Title/Abstract] OR "central african republic"[Title/Abstract] OR "Chad"[Title/Abstract] OR "Comoros"[Title/Abstract] OR "congo democratic republic of the"[Title/Abstract] OR "congo republic of the"[Title/Abstract]) OR "cote d ivoire"[Title/Abstract] OR "Djibouti"[Title/Abstract] OR "Egypt"[Title/Abstract] OR "equatorial guinea"[Title/Abstract] OR "Eritrea"[Title/Abstract] OR "Eswatini"[Title/Abstract] OR "Ethiopia"[Title/Abstract] OR "Gabon"[Title/Abstract] OR "Gambia"[Title/Abstract] OR "Ghana"[Title/Abstract] OR "Guinea"[Title/Abstract] OR "Guinea-Bissau"[Title/Abstract] OR "Kenya"[Title/Abstract] OR "Lesotho"[Title/Abstract] OR "Liberia"[Title/Abstract] OR "Libya"[Title/Abstract] OR "Madagascar"[Title/Abstract] OR "Malawi"[Title/Abstract] OR "Mali"[Title/Abstract] OR "Mauritania"[Title/Abstract] OR "Mauritius"[Title/Abstract] OR "Morocco"[Title/Abstract] OR "Mozambique"[Title/Abstract] OR "Namibia"[Title/Abstract] OR "Niger"[Title/Abstract] OR "Nigeria"[Title/Abstract] OR "Rwanda"[Title/Abstract] OR "sao tome and Principe"[Title/Abstract]) OR "Senegal"[Title/Abstract] OR "Seychelles"[Title/Abstract] OR "sierra leone"[Title/Abstract] OR "Somalia"[Title/Abstract] OR "south africa"[Title/Abstract] OR "south sudan"[Title/Abstract] OR "Sudan"[Title/Abstract] OR "Tanzania"[Title/Abstract] OR "Togo"[Title/Abstract] OR "Tunisia"[Title/Abstract] OR "Uganda"[Title/Abstract] OR "Zambia"[Title/Abstract] OR "Zimbabwe"[Title/Abstract])) | 426,523 |
| #2 | ((((Disease, Rheumatic Heart[Title/Abstract]) OR (Diseases, Rheumatic Heart[Title/Abstract])) OR (Heart Disease, Rheumatic[Title/Abstract])) OR (Heart Diseases, Rheumatic[Title/Abstract])) OR (Rheumatic Heart Diseases[Title/Abstract]) | 372 |
| #1 | ("prevalence"[Title/Abstract] OR "incidence"[Title/Abstract] OR "magnitude"[Title/Abstract] OR "Epidemiology"[Title/Abstract]) OR (Proportion[Title/Abstract]) | 2,374,099 |
