# Supplementary material for: Prevalence and pattern of rheumatic valvular heart disease in Africa: Systematic review and meta-analysis, 2015–2023, population based studies
Source: PLoS One. 2024 Jul 29;19(7):e0302636. doi: 10.1371/journal.pone.0302636 (PMC11285969; doi:10.1371/journal.pone.0302636)
Supplement: S2 Appendix — (DOCX) [file pone.0302636.s004.docx]

**Appendix 2. Quality assessment of included studies.**

Yes No Unclear N/A

1. Was the sample frame appropriate

to address the target population? □ □ □

1. Were study participant’s sample

in an appropriate way? □ □ □

1. Was the sample size adequate? □ □ □
2. Were the study subjects and

the setting described in detail? □ □ □

1. Was the data analysis conducted with

sufficient coverage of the identified sample?   □ □ □

1. Were valid methods used for

the identification of the condition?   □ □ □

1. Was the condition measured in

a standard, reliable way for all participants? □ □ □

1. Was there appropriate statistical analysis   □ □ □
2. Was the response rate adequate, and if not, was

the low response rate managed appropriately? □ □ □

Based on these criteria, the quality score for the included studies was given in the following table

**Table 1**. JBI Critical Appraisal Checklist for included Prevalence Studies

| **First Author name, Publication years** | **Criteria and corresponding scores** | | | | | | | | | **All over score** |
| --- | --- | --- | --- | --- | --- | --- | --- | --- | --- | --- |
|  | **1** | **2** | **3** | **4** | **5** | **6** | **7** | **8** | **9** |  |
| Tadesse Gemechu et al(2017) | 1 | 1 | 1 | 1 | 1 | 1 | 1 | 1 | 1 | 9 |
| Sulafa Ali et al  (2017) | 0 | 1 | 1 | 1 | 1 | 1 | 0 | 1 | 1 | 7 |
| Amy Scheel et al  (2018) | 1 | 1 | 1 | 1 | 1 | 1 | 1 | 1 | 0 | 8 |
| Mark E Engel et al  (2015) | 1 | 1 | 1 | 1 | 1 | 1 | 1 | 1 | 0 | 8 |
| Mark E Engel et al  (2015) | 1 | 1 | 1 | 1 | 1 | 1 | 1 | 1 | 0 | 8 |
| Mark E Engel et al  (2015) | 1 | 1 | 1 | 1 | 1 | 1 | 1 | 1 | 0 | 8 |
| Mark E Engel et al  (2015) | 1 | 1 | 1 | 1 | 1 | 1 | 1 | 1 | 0 | 8 |
| Ahmed Ali et al (2023) | 1 | 1 | 1 | 1 | 1 | 1 | 1 | 1 | 0 | 8 |
| Ekanem N. Ekure(  2019) | 1 | 1 | 1 | 1 | 1 | 1 | 1 | 1 | 1 | 9 |
| Aliou Alassane et al(2015) | 1 | 1 | 1 | 1 | 1 | 1 | 1 | 1 | 1 | 9 |
| Dr.Hailu Abera et al(2016) | 1 | 1 | 1 | 1 | 1 | 1 | 1 | 1 | 1 | 9 |
| Dejuma Yadeta et al  (2016) | 1 | 1 | 1 | 1 | 1 | 1 | 1 | 1 | 1 | 9 |
| J.mucumisti et al(2017) |  |  |  |  |  |  |  |  | 0 |  |
| John Musuku et al(2018) | 1 | 1 | 1 | 1 | 1 | 1 | 1 | 1 | 1 | 9 |
| AmySimsSanyahumbi etal(2016) |  |  |  |  |  |  |  |  |  |  |
| ParvinaTitusKazahura et al(2021) | 1 | 1 | 1 | 1 | 1 | 1 | 1 | 1 | 1 | 9 |
| Sulafa Ali et al(2018) |  |  |  |  |  |  |  |  |  |  |
| Sulafa Ali et al(2018) |  |  |  |  |  |  |  |  |  |  |
| Sulafa Ali et al(2018) | 1 | 1 | 1 | 1 | 0 | 1 | 1 | 1 | 1 | 8 |
| Sulafa Ali et al(2022) |  |  |  |  |  |  |  |  |  |  |
| Esin Nkereuwem et al(2020) |  |  |  |  |  |  |  |  |  |  |
| Ujuanbi A. et al(2019) | 1 | 1 | 0 | 1 | 1 | 1 | 1 | 1 | 1 | 8 |
|  |  |  |  |  |  |  |  |  |  |  |
