# Supplementary material for: Prevalence and pattern of rheumatic valvular heart disease in Africa: Systematic review and meta-analysis, 2015–2023, population based studies
Source: PLoS One. 2024 Jul 29;19(7):e0302636. doi: 10.1371/journal.pone.0302636 (PMC11285969; doi:10.1371/journal.pone.0302636)
Supplement: Attachment — Submitted filename: Prevalence and Pattern of RHD Review.docx [file pone.0302636.s005.docx]

Prevalence and Pattern of Rheumatic valvular Heart Disease in Africa Systematic Review and Meta-Analysis, 2015-2023, Population-Based Studies

The above titled manuscript general comments as stated below:

1. The manuscript requires a fundamental review of the sentences. There are many incomplete statements, typographical errors and unclear sentences that requires rephrasing.
2. The manuscript is submitted to PLOS ONE and not BMJ
3. The review and possible explanation given to some of the studies are not adequate. For example, explanation for differences in prevalence of rheumatic heart disease (line 227-28): what is it about the study duration? How cases where identified-You had earlier said all used echocardiography, so what ways where they identified?
4. The first paragraph in the discussion is not required.

Some of the poorly written sentences

Line 29 (result) : Out of which 18 articles

Line 32: with mitral valve regurgitation is

Line 44: WHO regions

Line 48: called for

Line 63: spelling through

Line 66: of in

5. Do not leave empty spaces in the tables. If no content put –
6. How did you arrive at the 14 studies discussed from the list in table 1?
7. Line 243; explanation for no gender disparity is unclear
8. No discussion on the publication bias.
9. Conclusion from the review?
10. Exclusion of studies written in other languages a limitation to results.
