# Supplementary material for: Prevalence and pattern of rheumatic valvular heart disease in Africa: Systematic review and meta-analysis, 2015–2023, population based studies
Source: PLoS One. 2024 Jul 29;19(7):e0302636. doi: 10.1371/journal.pone.0302636 (PMC11285969; doi:10.1371/journal.pone.0302636)
Supplement: Attachment — Submitted filename: Response to Reviewers.docx [file pone.0302636.s006.docx]

**Rebuttal letter**

Dear editor, and reviewers.

We would like to thank you for giving us the opportunity to submit a revised version of our manuscript titled “Prevalence and Pattern of Rheumatic Valvular Heart Disease in Africa: Systematic Review and Meta-Analysis, 2015-2023, Population-Based Studies." We are too grateful to you and the reviewers for taking the time and effort to read our manuscript and offer insightful comments. Based on these comments and suggestions, we have made careful modifications to the original manuscript. Your informed and helpful feedback helped us think of potential changes for the current version. We believe the manuscript has improved and hope that it meets your high standards. The authors welcome further constructive comments, if any. Please see below for a point-by-point response to comments and concerns. Comments are shown in black, followed by our answers and responses in dark blue. The changes are shown in the marked copy of the manuscript (highlighted in blue).Best regards,

**Academic Editor’s Comment**

Dear Dr. Abdu

Thank you for submitting your manuscript to PLOS ONE. After careful consideration, we feel that it has merit but does not fully meet PLOS ONE’s publication criteria as it currently stands. Therefore, we invite you to submit a revised version of the manuscript that addresses the points raised during the review process. Please revise the manuscript based on the feedback from reviewers.

Please include the following items when submitting your revised manuscript:

- A rebuttal letter that responds to each point raised by the academic editor and reviewer(s). You should upload this letter as a separate file labeled 'Response to Reviewers'.
- A marked-up copy of your manuscript that highlights changes made to the original version. You should upload this as a separate file labeled 'Revised Manuscript with Track Changes'.
- An unmarked version of your revised paper without tracked changes. You should upload this as a separate file labeled 'Manuscript'.

If you would like to make changes to your financial disclosure, please include your updated statement in your cover letter. Guidelines for resubmitting your figure files are available below the reviewer comments at the end of this letter.

If applicable, we recommend that you deposit your laboratory protocols in [protocols.io](http://protocols.io/) to enhance the reproducibility of your results

Journal requirements:

When submitting your revision, we need you to address these additional requirements.

1. Please ensure that your manuscript meets PLOS ONE's style requirements, including those for file naming.

**Response:** We agree with this and have incorporated your suggestion throughout the revised manuscript.

2. We noticed you have some minor occurrence of overlapping text with the following previous publication(s), which needs to be addressed: https://apps.who.int/gb/ebwha/pdf_files/WHA71/A71_25-en.pdf. In your revision ensure you cite all your sources (including your own works), and quote or rephrase any duplicated text outside the methods section. Further consideration is dependent on these concerns being addressed

**Response:** Thank you for this suggestion. We made every effort to thoroughly rephrase any redundant content, cite our sources, and copyedit our work to remove any instances of overlapping text usage.

3. In this instance it seems there may be acceptable restrictions in place that prevent the public sharing of your minimal data. However, in line with our goal of ensuring long-term data availability to all interested researchers, PLOS’ Data Policy states that authors cannot be the sole named individuals responsible for ensuring data access (<http://journals.plos.org/plosone/s/data-availability#loc-acceptable-data-sharing-methods>). Data requests to a non-author institutional point of contact, such as a data access or ethics committee, helps guarantee long term stability and availability of data. Providing interested researchers with a durable point of contact ensures data will be accessible even if an author changes email addresses, institutions, or becomes unavailable to answer requests. Before we proceed with your manuscript, please also provide non-author contact information (phone/email/hyperlink) for a data access committee, ethics committee, or other institutional body to which data requests may be sent. If no institutional body is available to respond to requests for your minimal data, please consider if there any institutional representatives who did not collaborate in the study, and are not listed as authors on the manuscript, who would be able to hold the data and respond to external requests for data access? If so, please provide their contact information (i.e., email address). Please also provide details on how you will ensure persistent or long-term data storage and availability.

**Response:** Thank you for highlighting this. Since all of the data we gathered was compiled from previously published research, all are available freely in their respective journals for anyone to access. Moreover all relevant data are within the manuscript and its Supporting Information files. As such, we ensure continuous and unimpeded access to the study data information.

4. Please include captions for your Supporting Information files at the end of your manuscript, and update any in-text citations to match accordingly. Please see our Supporting Information guidelines for more information: <http://journals.plos.org/plosone/s/supporting-information>.

Response: I appreciate you bringing this to our attention. We add captions for our supporting information files at the end of our manuscript and update in-text citations to match accordingly.

**Reviewers' comments:**

[NOTE: If reviewer comments were submitted as an attachment file, they will be attached to this email and accessible via the submission site. Please log into your account, locate the manuscript record, and check for the action link "View Attachments". If this link does not appear, there are no attachment files.]

While revising your submission, please upload your figure files to the Preflight Analysis and Conversion Engine (PACE) digital diagnostic tool, https://pacev2.apexcovantage.com/. PACE helps ensure that figures meet PLOS requirements. To use PACE, you must first register as a user. Registration is free. Then, login and navigate to the UPLOAD tab, where you will find detailed instructions on how to use the tool. If you encounter any issues or have any questions when using PACE, please email PLOS at figures@plos.org. Please note that Supporting Information files do not need this step.

**Response:** The authors thank the reviewer for this kind deep insight. Before submitting the paper to the journal once more, we upload the graph to the Preflight Analysis and Conversion Engine (PACE) and replace the original graph with the converted paced graph in the revised manuscript.

1. The manuscript requires a fundamental review of the sentences. There are many incomplete statements, typographical errors and unclear sentences that requires rephrasing.

**Response:** We thank the reviewer for this good suggestion. We have carefully gone over the entire work, checking for typos, unclear sentences, and entire paragraphs. We made every effort to ensure that it was clear and concise.

2. The manuscript is submitted to PLOS ONE and not BMJ?

**Response:** We appreciate this comments. Indeed, we correct it in cover letter.

3. The review and possible explanation given to some of the studies are not adequate. For example, explanation for differences in prevalence of rheumatic heart disease (line 227-28): what is it about the study duration? How cases where identified-You had earlier said all used echocardiography, so what ways where they identified?

**Response:** Thanks for your kind question. We agree and have now included in the amended manuscript detailed explanations for the discrepancies in study duration, diagnosis methods, and prevalence of rheumatic heart disease where they were studied.

4. The first paragraph in the discussion is not required.

Some of the poorly written sentences

Line 29 (result) : Out of which 18 articles

Line 32: with mitral valve regurgitation is

Line 44: WHO regions

Line 48: called for

Line 63: spelling through

Line 66: of in

**Response:** Thank you for your comment. We agree and have now removed unnecessary sentence from the first paragraph of discussion in the revised manuscript. In order to be cohesive, we corrected each line by line as suggested by the reviewer and we have tried to correct grammatical errors throughout the entire manuscript.

5. Do not leave empty spaces in the tables. If no content put –

**Response:** Thank you for noticing. We have added hyphen (-) to empty spaces in the table

6. How did you arrive at the 14 studies discussed from the list in table 1?

**Response:** Thank you for your thorough review and salient observations. We have included the required explanation from the analysis using a forest plot in to the revised manuscript.

7. Line 243; explanation for no gender disparity is unclear

**Response:** Thank you for pointing this out. We agree with this comment, which has now been amended in the revised discussion in detail.

8. No discussion on the publication bias.

**Response:** The authors thank you for your interesting comment. We have add the text following your suggestion by discussing the publication bias as follows: “Finally, an additional limitation we encountered in our review was the potential presence of publication bias. To mitigate this bias, we conducted a non-parametric trim and fill analysis. Through this method, we estimated the number of missing studies and imputed three additional studies”.

9. Conclusion from the review?

**Response:** The authors would like to thank the reviewer for raising this point. In conclusion, we have made every effort to incorporate the necessary points into the revised manuscript. We believe these additions strengthen the overall quality and comprehensiveness of our work.

10. Exclusion of studies written in other languages a limitation to results.

**Response:** Thank you very much for your suggestion. We found your comment is helpful and has now been written clearly as suggested.
